# Supplementary material for: Optimal selection of COVID-19 vaccination sites in the Philippines at the municipal level
Source: PeerJ. 2022 Sep 30;10:e14151. doi: 10.7717/peerj.14151 (PMC9528907; doi:10.7717/peerj.14151)
Supplement: Supplemental Information 1 — Figure S1 map credit: Marvin A. Sinag, https://commons.wikimedia.org/wiki/File:Ph_map_of_san_juan_batangas.png [file peerj-10-14151-s001.docx]

**Supplementary File for the paper “Optimal Selection of COVID-19 Vaccination Sites at the Municipal Level”**

**Kurt Izak M. Cabanilla, Erika Antonette T. Enriquez, Renier G. Mendoza, and Victoria May P. Mendoza**

Institute of Mathematics, University of the Philippines Diliman,

Quezon City, Philippines

San Juan is a municipality in the province of Batangas, located in the Luzon Island of the Philippines. It consists of 42 barangays. A map of San Juan, Batangas is shown in Figure 1.


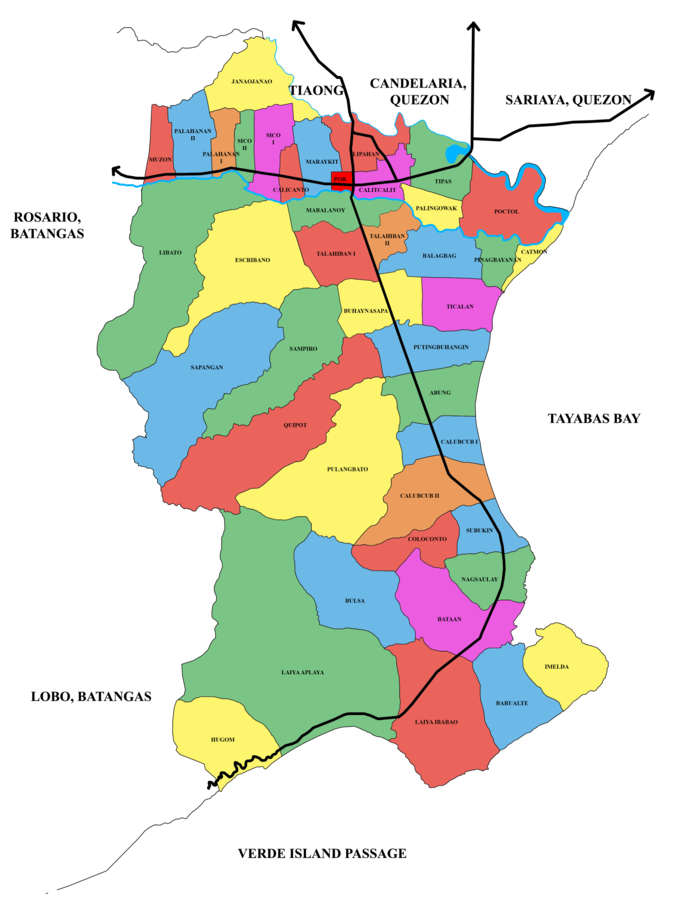


**Figure 1.** A map showing the barangays of San Juan, Batangas, the Philippines. The image was made by Marvin Sinag in 2019 and was obtained from [1].

The population data were collected from the website of the Philippine Department of Health [2]. The number of COVID-19 confirmed cases per barangay in San Juan, Batangas was obtained from the Facebook page of the San Juan local government unit [3]. These data are summarized in Table I.

The list of hospitals and schools was manually generated from the directories of the Philippine Department of Health [4], Philippine Commission on Higher Education [5], and the Philippine Department of Education [6]. These data are summarized in Table II.

The coordinates of the barangay halls and vaccination sites were collected with the help of the OSM package in Python and Google maps.

The complete list of barangays and their assigned vaccination site is given in Table III.

| **Table I: All the barangays in San Juan, Batangas**  **with their projected population in 2021, number of confirmed COVID-19 cases as of May 31, 2021, and coordinates** | | | | |
| --- | --- | --- | --- | --- |
| **NAME** | **POPULATION** | **NO. OF CASES** | **LATITUDE** | **LONGITUDE** |
| Abung | 2,444 | 14 | 13.7685 | 121.4156 |
| Balagbag | 2,929 | 7 | 13.8032 | 121.4165 |
| Barualte | 1,933 | 2 | 13.6874 | 121.4389 |
| Bataan | 2,204 | 3 | 13.6964 | 121.4333 |
| Buhay Na Sapa | 5,909 | 26 | 13.7905 | 121.4081 |
| Bulsa | 2,492 | 3 | 13.7027 | 121.4003 |
| Calicanto | 2,257 | 13 | 13.8258 | 121.3821 |
| Calitcalit | 5,255 | 59 | 13.8268 | 121.4111 |
| Calubcub I | 2,545 | 13 | 13.7534 | 121.4211 |
| Calubcub II | 4,071 | 9 | 13.7421 | 121.4262 |
| Catmon | 1,636 | 0 | 13.8066 | 121.4503 |
| Coloconto | 874 | 8 | 13.7228 | 121.4192 |
| Escribano | 3,755 | 8 | 13.8122 | 121.3644 |
| Hugom | 1,820 | 7 | 13.6631 | 121.3731 |
| Imelda (Tubog) | 1,106 | 3 | 13.6900 | 121.4510 |
| Janaojanao | 1,743 | 1 | 13.8539 | 121.3677 |
| Laiya-Aplaya | 6,927 | 14 | 13.6762 | 121.3876 |
| Laiya-Ibabao | 5,195 | 11 | 13.6807 | 121.4161 |
| Libato | 4,737 | 10 | 13.8156 | 121.3511 |
| Lipahan | 5,052 | 42 | 13.8339 | 121.4056 |
| Mabalanoy | 4,423 | 38 | 13.8183 | 121.3975 |
| Maraykit | 4,123 | 42 | 13.8258 | 121.3872 |
| Muzon | 1,775 | 11 | 13.8281 | 121.3409 |
| Nagsaulay | 3,004 | 21 | 13.7117 | 121.4404 |
| Palahanan I | 970 | 2 | 13.8297 | 121.3608 |
| Palahanan II | 3,730 | 13 | 13.8303 | 121.3523 |
| Palingowak | 1,883 | 7 | 13.8217 | 121.4136 |
| Pinagbayanan | 1,739 | 3 | 13.8008 | 121.4367 |
| Poblacion | 3,785 | 76 | 13.8276 | 121.3947 |
| Poctol | 2,939 | 16 | 13.8205 | 121.4408 |
| Pulangbato | 3,192 | 1 | 13.7472 | 121.3967 |
| Putingbuhangin | 2,873 | 22 | 13.7819 | 121.4231 |
| Quipot | 3,839 | 6 | 13.7709 | 121.3860 |
| Sampiro | 3,635 | 7 | 13.7866 | 121.3924 |
| Sapangan | 3,391 | 15 | 13.7840 | 121.3582 |
| Sico I | 2,280 | 19 | 13.8282 | 121.3732 |
| Sico II | 1,269 | 8 | 13.8295 | 121.3632 |
| Subukin | 1,886 | 2 | 13.7298 | 121.4380 |
| Talahiban I | 2,588 | 3 | 13.8089 | 121.3931 |
| Talahiban II | 1,501 | 3 | 13.8095 | 121.4039 |
| Ticalan | 2,111 | 6 | 13.7924 | 121.4268 |
| Tipaz | 3,432 | 5 | 13.8317 | 121.4283 |
| **TOTAL** | 125,252 | 579 |  |  |

| **Table II: Names of the vaccination sites in San Juan, Batangas**  **with their corresponding site type, barangay location, and coordinates** | | | | |
| --- | --- | --- | --- | --- |
| **TYPE** | **NAME** | **BARANGAY** | **LATITUDE** | **LONGITUDE** |
| **Hospital** | San Juan Rural Health Unit II | Buhay Na Sapa | 13.7913 | 121.4076 |
|  | San Juan Doctors Hospital, Inc. | Calicalit | 13.8265 | 121.4100 |
|  | San Juan Rural Health Unit I | Poblacion | 13.8248 | 121.3962 |
|  | Divine Care Hospital Multi Services Corp. | Sico II | 13.8290 | 121.3674 |
|  | San Juan District Hospital | Talahiban II | 13.8096 | 121.4007 |
| **Elementary**  **School** | Abung Elementary School | Abung | 13.7688 | 121.4155 |
|  | Balagbag Elementary School | Balagbag | 13.8033 | 121.4186 |
|  | Paaralang Elementarya Ng Barualte | Barualte | 13.6870 | 121.4343 |
|  | Paaralang Elementarya ng Bataan | Bataan | 13.6961 | 121.4329 |
|  | Paaralang Elementarya Ng Buhaynasapa | Buhay Na Sapa | 13.7909 | 121.4121 |
|  | Paaralang Elementarya ng Bulsa | Bulsa | 13.7039 | 121.4016 |
|  | Calitcalit Elementary School | Calicalit | 13.8281 | 121.4108 |
|  | Paaralang Elementarya ng Marcal | Calicanto | 13.8255 | 121.3822 |
|  | Calubcub I Elementary School | Calubcub I | 13.7536 | 121.4188 |
|  | Calubcub II Elementary School | Calubcub II | 13.7400 | 121.4256 |
|  | Catmon Elementary School | Catmon | 13.8072 | 121.4511 |
|  | Coloconto Elementary School | Coloconto | 13.7223 | 121.4193 |
|  | Paaralang Elementarya ng Escribano | Escribano | 13.8129 | 121.3645 |
|  | Hugom Elementary School | Hugom | 13.6631 | 121.3730 |
|  | Imelda Elementary School | Imelda | 13.6907 | 121.4521 |
|  | Paaralang Elementarya ng Janaojanao | Janaojanao | 13.8533 | 121.3622 |
|  | Aplaya Elementary School | Laiya-Aplaya | 13.6757 | 121.3978 |
|  | Wenceslao I. Llana Memorial School | Laiya-Aplaya | 13.6889 | 121.3943 |
|  | Laiya Elementary School | Laiya-Ibabao | 13.6818 | 121.4157 |
|  | Paaralang Elementarya ng Libato | Libato | 13.8047 | 121.3468 |
|  | San Juan East Central School | Lipahan | 13.8305 | 121.3970 |
|  | Mabalanoy Elementary School | Mabalanoy | 13.8189 | 121.3855 |
|  | Paaralang Elementarya ng Muzon | Muzon | 13.8341 | 121.3422 |
|  | Nagsaulay Elementary School | Nagsaulay | 13.7117 | 121.4405 |
|  | Palahanan Elementary School | Palahanan II | 13.8294 | 121.3522 |
|  | Palingowak Elementary School | Palingowak | 13.8237 | 121.4179 |
|  | Pinagbayanan Elementary School | Pinagbayanan | 13.8014 | 121.4368 |
|  | San Juan West Central School | Poblacion | 13.8273 | 121.3945 |
|  | Paaralang Elementarya ng Poctol | Poctol | 13.8224 | 121.4409 |
|  | Paaralang Elementarya ng Pulangbato | Pulangbato | 13.7470 | 121.3974 |
|  | Paaralang Elementarya ng Putingbuhangin | Putingbuhangin | 13.7827 | 121.4228 |
|  | Paaralang Elementarya ng Calabasahan | Quipot | 13.7471 | 121.3569 |
|  | Paaralang Elementarya ng Quipot | Quipot | 13.7778 | 121.3985 |
|  | Paaralang Elementarya ng Sampiro | Sampiro | 13.7842 | 121.3853 |
|  | Libjo Sapangan Elementary School | Sapangan | 13.7695 | 121.3651 |
|  | Paaralang Elementarya ng Sapangan | Sapangan | 13.7845 | 121.3587 |
|  | Paaralang Elementarya ng Pal-Sico | Sico II | 13.8292 | 121.3663 |
|  | Subukin Elementary School | Subukin | 13.7310 | 121.4372 |
|  | Paaralang Elementarya ng Talahiban 1.0 | Talahiban I | 13.8088 | 121.3909 |
|  | Talahiban 2.0 Elementary School | Talahiban II | 13.8086 | 121.4060 |
|  | Paaralang Elementarya ng Ticalan | Ticalan | 13.7924 | 121.4270 |
|  | Paaralang Elementarya ng Tipas | Tipaz | 13.8313 | 121.4304 |
| **Junior High**  **School** | Buhaynasapa National High School | Buhay Na Sapa | 13.7915 | 121.4119 |
|  | Angeles Luistro NHS | Bulsa | 13.7107 | 121.4009 |
|  | Calubcub 1.0 National High School | Calubcub I | 13.7535 | 121.4231 |
|  | Aplaya National High School | Laiya-Aplaya | 13.6766 | 121.3875 |
|  | Laiya National High School | Laiya-Ibabao | 13.6818 | 121.4157 |
|  | Lipahan National High School | Lipahan | 13.8343 | 121.3965 |
|  | Don Leon Mercado Sr. MNHS | Mabalanoy | 13.8149 | 121.3987 |
|  | Nagsaulay National High School | Nagsaulay | 13.7153 | 121.4400 |
|  | Palahanan National High School | Palahanan II | 13.8298 | 121.3523 |
|  | Sampiro National High School | Sampiro | 13.7668 | 121.3699 |
|  | Pacita Ramos Mendoza MNHS | Sapangan | 13.7848 | 121.3587 |
|  | Sico 1.0 National High School | Sico I | 13.8277 | 121.3732 |
|  | Tipas National High School | Tipaz | 13.8321 | 121.4286 |
| **Senior High**  **School** | San Juan Senior High School | Lipahan | 13.8313 | 121.3969 |
|  | Joseph Marello Institute | Poblacion | 13.8277 | 121.3944 |
| **University** | Batangas Eastern Colleges | Poblacion | 13.8291 | 121.3955 |
|  | Batangas State University - San Juan Campus | Talahiban II | 13.8023 | 121.4033 |
| **Church** | San Juan Nepomuceno Church | Poblacion | 13.8281 | 121.3947 |

| **Table III. Vaccination center assignments of all the barangays for the four different numbers of vaccination sites.** | | | | | | | | |
| --- | --- | --- | --- | --- | --- | --- | --- | --- |
| **Barangay** | **One Site** | | **Two Sites** | | **Three Sites** | | **Four Sites** | |
|  | **Vaccination Site** | **Distance (m)** | **Vaccination Site** | **Distance**  **(m)** | **Vaccination Site** | **Distance**  **(m)** | **Vaccination Site** | **Distance**  **(m)** |
| Abung | Don Leon Mercado Sr. MNHS | 5390.984 | San Juan Rural Health Unit I | 6692.139 | San Juan Rural Health Unit II | 2577 | San Juan Rural Health Unit II | 2577 |
| Balagbag | Don Leon Mercado Sr. MNHS | 2763.148 | San Juan Rural Health Unit I | 4064.303 | San Juan Rural Health Unit II | 2314.88 | San Juan Rural Health Unit II | 2314.88 |
| Barualte | Don Leon Mercado Sr. MNHS | 17369.653 | Paaralang Elementarya ng Bataan | 2693.793 | Laiya National High School | 2878.46 | Laiya Elementary School | 2878.46 |
| Bataan | Don Leon Mercado Sr. MNHS | 14675.86 | Paaralang Elementarya ng Bataan | 0 | Laiya National High School | 2182.96 | Laiya Elementary School | 2182.96 |
| Buhay Na Sapa | Don Leon Mercado Sr. MNHS | 2813.989 | San Juan Rural Health Unit I | 4115.144 | San Juan Rural Health Unit II | 0 | San Juan Rural Health Unit II | 0 |
| Bulsa | Don Leon Mercado Sr. MNHS | 15494.69 | Paaralang Elementarya ng Bataan | 4552.727 | Laiya National High School | 4737.4 | Laiya Elementary School | 4737.4 |
| Calicanto | Don Leon Mercado Sr. MNHS | 3151.142 | San Juan Rural Health Unit I | 1857.736 | San Juan Rural Health Unit I | 1857.74 | Sico 1.0 National High School | 1176.85 |
| Calitcalit | Don Leon Mercado Sr. MNHS | 2810.548 | San Juan Rural Health Unit I | 1684.927 | San Juan Rural Health Unit I | 1684.93 | San Juan Doctors Hospital, Inc. | 143.837 |
| Calubcub I | Don Leon Mercado Sr. MNHS | 7189.882 | Paaralang Elementarya ng Bataan | 7485.978 | San Juan Rural Health Unit II | 4375.89 | San Juan Rural Health Unit II | 4375.89 |
| Calubcub II | Don Leon Mercado Sr. MNHS | 8908.374 | Paaralang Elementarya ng Bataan | 6221.284 | San Juan Rural Health Unit II | 6094.39 | San Juan Rural Health Unit II | 6094.39 |
| Catmon | Don Leon Mercado Sr. MNHS | 6905.325 | San Juan Rural Health Unit I | 7447.483 | San Juan Rural Health Unit II | 6457.06 | San Juan Doctors Hospital, Inc. | 5906.39 |
| Coloconto | Don Leon Mercado Sr. MNHS | 12431.169 | Paaralang Elementarya ng Bataan | 5979.296 | Laiya National High School | 7047.15 | Laiya Elementary School | 7047.15 |
| Escribano | Don Leon Mercado Sr. MNHS | 5636.594 | San Juan Rural Health Unit I | 5269.217 | San Juan Rural Health Unit I | 5269.22 | Sico 1.0 National High School | 2669.99 |
| Hugom | Don Leon Mercado Sr. MNHS | 22497.002 | Paaralang Elementarya ng Bataan | 7821.142 | Laiya National High School | 5638.18 | Laiya Elementary School | 5638.18 |
| Imelda | Don Leon Mercado Sr. MNHS | 19029.53 | Paaralang Elementarya ng Bataan | 4353.67 | Laiya National High School | 4538.34 | Laiya Elementary School | 4538.34 |
| Janaojanao | Don Leon Mercado Sr. MNHS | 7203.94 | San Juan Rural Health Unit I | 5910.534 | San Juan Rural Health Unit I | 5910.53 | Sico 1.0 National High School | 3311.31 |
| Laiya-Aplaya | Don Leon Mercado Sr. MNHS | 20751.808 | Paaralang Elementarya ng Bataan | 6075.948 | Laiya National High School | 3892.99 | Laiya Elementary School | 3892.99 |
| Laiya-Ibabao | Don Leon Mercado Sr. MNHS | 17023.915 | Paaralang Elementarya ng Bataan | 2348.055 | Laiya National High School | 165.096 | Laiya Elementary School | 165.096 |
| Libato | Don Leon Mercado Sr. MNHS | 6549.993 | San Juan Rural Health Unit I | 6182.616 | San Juan Rural Health Unit I | 6182.62 | Sico 1.0 National High School | 3583.39 |
| Lipahan | Don Leon Mercado Sr. MNHS | 3533.293 | San Juan Rural Health Unit I | 2381.999 | San Juan Rural Health Unit I | 2382 | San Juan Doctors Hospital, Inc. | 897.041 |
| Mabalanoy | Don Leon Mercado Sr. MNHS | 469.022 | San Juan Rural Health Unit I | 832.133 | San Juan Rural Health Unit I | 832.133 | San Juan Doctors Hospital, Inc. | 2197.69 |
| Maraykit | Don Leon Mercado Sr. MNHS | 2435.774 | San Juan Rural Health Unit I | 1142.368 | San Juan Rural Health Unit I | 1142.37 | Sico 1.0 National High School | 1456.86 |
| Muzon | Don Leon Mercado Sr. MNHS | 7273.1 | San Juan Rural Health Unit I | 5979.694 | San Juan Rural Health Unit I | 5979.69 | Sico 1.0 National High School | 3380.47 |
| Nagsaulay | Don Leon Mercado Sr. MNHS | 12538.092 | Paaralang Elementarya ng Bataan | 2137.768 | Laiya National High School | 4320.73 | Laiya Elementary School | 4320.73 |
| Palahanan I | Don Leon Mercado Sr. MNHS | 5200.798 | San Juan Rural Health Unit I | 3907.392 | San Juan Rural Health Unit I | 3907.39 | Sico 1.0 National High School | 1308.17 |
| Palahanan II | Don Leon Mercado Sr. MNHS | 6504.722 | San Juan Rural Health Unit I | 5211.316 | San Juan Rural Health Unit I | 5211.32 | Sico 1.0 National High School | 2612.09 |
| Palingowak | Don Leon Mercado Sr. MNHS | 2707.563 | San Juan Rural Health Unit I | 2452.859 | San Juan Rural Health Unit I | 2452.86 | San Juan Doctors Hospital, Inc. | 911.769 |
| Pinagbayanan | Don Leon Mercado Sr. MNHS | 5078.29 | San Juan Rural Health Unit I | 6379.445 | San Juan Rural Health Unit II | 4630.02 | San Juan Rural Health Unit II | 4630.02 |
| Poblacion | Don Leon Mercado Sr. MNHS | 1807.502 | San Juan Rural Health Unit I | 511.658 | San Juan Rural Health Unit I | 511.658 | San Juan Doctors Hospital, Inc. | 1889.99 |
| Poctol | Don Leon Mercado Sr. MNHS | 6240.627 | San Juan Rural Health Unit I | 5166.453 | San Juan Rural Health Unit I | 5166.45 | San Juan Doctors Hospital, Inc. | 3625.36 |
| Pulangbato | Don Leon Mercado Sr. MNHS | 10500.275 | Paaralang Elementarya ng Bataan | 10660.25 | San Juan Rural Health Unit II | 7686.29 | San Juan Rural Health Unit II | 7686.29 |
| Putingbuhangin | Don Leon Mercado Sr. MNHS | 5132.689 | San Juan Rural Health Unit I | 6433.844 | San Juan Rural Health Unit II | 2318.7 | San Juan Rural Health Unit II | 2318.7 |
| Quipot | Don Leon Mercado Sr. MNHS | 6892.508 | San Juan Rural Health Unit I | 8193.663 | San Juan Rural Health Unit II | 4078.52 | San Juan Rural Health Unit II | 4078.52 |
| Sampiro | Don Leon Mercado Sr. MNHS | 4411.659 | San Juan Rural Health Unit I | 5712.814 | San Juan Rural Health Unit II | 1726.99 | San Juan Rural Health Unit II | 1726.99 |
| Sapangan | Don Leon Mercado Sr. MNHS | 8166.059 | San Juan Rural Health Unit I | 9467.214 | San Juan Rural Health Unit II | 5724.42 | San Juan Rural Health Unit II | 5724.42 |
| Sico I | Don Leon Mercado Sr. MNHS | 3892.633 | San Juan Rural Health Unit I | 2599.227 | San Juan Rural Health Unit I | 2599.23 | Sico 1.0 National High School | 0 |
| Sico II | Don Leon Mercado Sr. MNHS | 4918.699 | San Juan Rural Health Unit I | 3625.293 | San Juan Rural Health Unit I | 3625.29 | Sico 1.0 National High School | 1026.07 |
| Subukin | Don Leon Mercado Sr. MNHS | 10459.058 | Paaralang Elementarya ng Bataan | 4216.802 | Laiya National High School | 6399.76 | Laiya Elementary School | 6399.76 |
| Talahiban I | Don Leon Mercado Sr. MNHS | 1288.43 | San Juan Rural Health Unit I | 2589.585 | San Juan Rural Health Unit I | 2589.58 | San Juan Rural Health Unit II | 3060.74 |
| Talahiban II | Don Leon Mercado Sr. MNHS | 821.825 | San Juan Rural Health Unit I | 2122.98 | San Juan Rural Health Unit I | 2122.98 | San Juan Rural Health Unit II | 2967.37 |
| Ticalan | Don Leon Mercado Sr. MNHS | 5144.105 | San Juan Rural Health Unit I | 6445.26 | San Juan Rural Health Unit II | 2330.12 | San Juan Rural Health Unit II | 2330.12 |
| Tipaz | Don Leon Mercado Sr. MNHS | 4764.887 | San Juan Rural Health Unit I | 3639.266 | San Juan Rural Health Unit I | 3639.27 | San Juan Doctors Hospital, Inc. | 2098.18 |

*References:*

1. <https://commons.wikimedia.org/wiki/File:Ph_map_of_san_juan_batangas.png>
2. <https://doh.gov.ph/publications>
3. <https://www.facebook.com/lgusanjuanbatangas>
4. <https://nhfr.doh.gov.ph/rfacilities2list.php?pageno=1&t=rfacilities2&recperpage=ALL>
5. <https://ched.gov.ph/list-higher-education-institutions/>
6. <https://www.deped.gov.ph/k-to-12/senior-high-school/list-of-senior-high-schools/>
